# 4556 Gender homophily in translational collaborations; a network analysis study of investigators at one academic medical center

**DOI:** 10.1017/cts.2020.357

**Published:** 2020-07-29

**Authors:** Reza Yousefi Nooraie, Elizabeth Wayman, Ann Dozier

**Affiliations:** 1 University of Rochester Medical Center; 2 University of Rochester

## Abstract

OBJECTIVES/GOALS: Collaborations are at the core of translational science and team science. Differences by gender have been identified in various research contexts from recruitment to retention to promotion. This study assesses the relational associations of translational collaborations, and what role of gender. METHODS/STUDY POPULATION: In 2011 and 2013, clinical and basic sciences investigators at University of Rochester School of Medicine and Dentistry responded to an online survey nominating their research collaborators. Two study years were merged, and name lists were transformed into a collaboration network. Departments were classified into basic sciences (e.g. biochemistry) and clinical (e.g. urology). If respondent and partner were affiliated to different department classes, the collaboration was defined as translational. Multi-level GLM models were developed to assess the associates of the likelihood of translational vs. within discipline collaborations. Partner nominations were nested in respondents. RESULTS/ANTICIPATED RESULTS: 202 respondents were included in the multi-level GLM models. A collaboration was more likely to be translational if the respondent shared more collaborators with the partner (OR:1.13), and respondent was a central actor in collaboration network (OR: 1.2). Translational collaborations were less likely to be reported by clinicians (OR: 0.25). In the model to assess gender match, a collaboration was more likely to be translational if the respondent was male, and nominated a male partner. For both genders, collaboration with a partner of the opposite gender was more likely to be translational if respondent had more shared collaborators with the partner. DISCUSSION/SIGNIFICANCE OF IMPACT: Translational collaborations happen in teams. Gender homophily exits in translational collaborations, and is reduced by shared collaborators; implying the effect of personal connections and community membership. Community-building interventions may increase diversity in translational collaborations.

